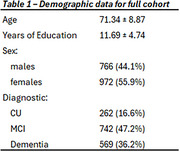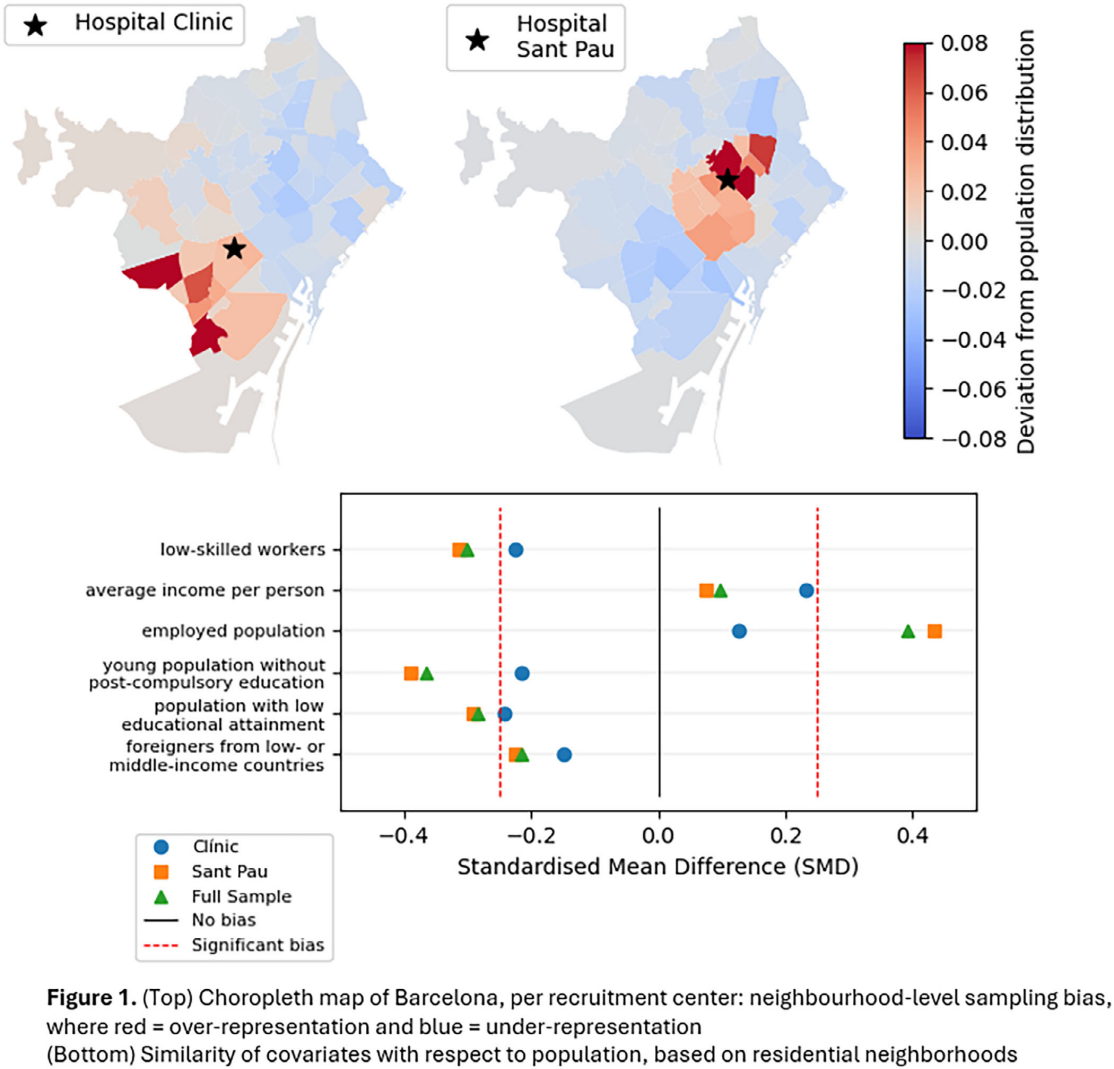# Representativeness and bias of neighborhood‐level determinants in UBRAIN: a multi‐site Aging and Dementia cohort

**DOI:** 10.1002/alz70861_108202

**Published:** 2025-12-23

**Authors:** Alex López, Carmen M Colceriu, Pablo Aguilar, Eleni Palpatzis, Adrià Tort‐Merino, Maria Franquesa‐Mullerat, Sara E Zsadanyi, Antonia Valentín, Sami Petricola, Juan Fortea, Alberto Lleó, Neus Falgàs Martínez, Raquel Sánchez‐Valle, Alexandre Bejanin, Eider M Arenaza‐Urquijo

**Affiliations:** ^1^ Global Health Institute Barcelona (ISGlobal), Barcelona Spain; ^2^ University of Pompeu Fabra (UPF), Barcelona Spain; ^3^ Sant Pau Memory Unit, Department of Neurology, Hospital de la Santa Creu i Sant Pau, Institut d'Investigació Biomèdica Sant Pau (IIB SANT PAU), Facultad de Medicina ‐ Universitat Autònoma de Barcelona, Barcelona Spain; ^4^ Alzheimer's Disease and Other Cognitive Disorders Unit, Neurology Department, Hospital Clinic, Barcelona Spain; ^5^ Centro de Investigación Biomédica en Red de Fragilidad y Envejecimiento Saludable (CIBERFES), Madrid Spain

## Abstract

**Background:**

A multi‐site cohort in Barcelona, Spain ‐ UBRAIN ‐ was used to analyze the impact of neighborhood‐level social determinants on aging‐ and dementia‐related outcomes, using Geographic Information Systems (GIS) data. This work focuses on analyzing the representativeness and social disparity‐related bias of the cohort to reveal practical challenges of leveraging GIS data from hospital‐led research cohorts in the study of social determinants of health.

**Methods:**

We included 2124 older adults and patients ‐ 16.6% cognitively unimpaired, 47.2% mild cognitive impairment, and 36.2% dementia (62.4% Alzheimer’s Disease, 14.8% Frontotemporal dementia, 13.2% Lewy body, other 9.6%) recruited at Clinic and Sant Pau Hospitals in Barcelona (Spain) (Table 1). We compared the cohort’s residential distribution to the population distribution data (Barcelona Open Data) using global Moran’s I applied to the neighborhood‐level sample‐to‐population ratio. We quantified bias in multiple socioeconomic indicators via standardized mean differences (SMD) between sample‐weighted and population‐weighted means, with |SMD| ≥ 0.25 denoting strong sampling bias. All analyses were also performed stratifying by sex.

**Results:**

We find that our sample’s spatial distribution significantly differs from the population distribution in Barcelona (Moran’s I=0.451, *p* <0.001), mostly centering around the recruitment sites (Figure 1 ‐ top). The resulting sample lives in neighborhoods with higher employment rates, lower rates of low educational attainment, and fewer low‐skilled workers (Figure 1 ‐ bottom). The magnitude of these SMDs was virtually independent of sex (maximum |ΔSMD| < 0.04). Stratifying by hospital showed a reduced socioeconomic bias in the sample from hospital Clínic.

**Conclusion:**

Our analysis of the UBRAIN cohort reveals a significant spatial and socioeconomic bias, with participants disproportionately residing in neighborhoods with few low‐skilled workers, high employment rates, and a highly educated population. These differences in representativeness should be taken into account when interpreting results from such cohorts, especially in the context of urban and social determinants of health. Efforts should be made to include participants from neighborhoods with lower socioeconomic status.